# Spiegelmer-Based Sandwich Assay for Cardiac Troponin I Detection

**DOI:** 10.3390/ijms21144963

**Published:** 2020-07-14

**Authors:** Zoltán János Tolnai, Judit András, Zsuzsanna Szeitner, Krisztina Percze, László Ferenc Simon, Róbert E. Gyurcsányi, Tamás Mészáros

**Affiliations:** 1 Department of Medical Chemistry, Molecular Biology and Pathobiochemistry, Semmelweis University, Tűzoltó u. 37-47., H-1094 Budapest, Hungary; tolnai.zoltan@med.semmelweis-univ.hu (Z.J.T.); andrasjudit@yahoo.com (J.A.); szeitner.zsuzsanna@med.semmelweis-univ.hu (Z.S.); percze.krisztina@med.semmelweis-univ.hu (K.P.); 2 BME “Lendület” Chemical Nanosensors Research Group, Department of Inorganic and Analytical Chemistry, Budapest University of Technology and Economics, Szt. Gellért tér 4, H-1111 Budapest, Hungary; simon.laszlo@mail.bme.hu (L.F.S.); robertgy@mail.bme.hu (R.E.G.)

**Keywords:** spiegelmer, troponinI, sandwich assay

## Abstract

Two subunits of the ternary troponin complex, I and C, have cardiac muscle specific isoforms, and hence could be applied as highly-selective markers of acute coronary syndrome. We aimed at paving the way for the development of a robust cardiac troponin I-detecting sandwich assay by replacing antibodies with nuclease resistant aptamer analogues, so-called spiegelmers. To complement the previously generated spiegelmers that were specific for the N-terminus of cTnI, spiegelmers were selected for an amino acid stretch in the proximity of the C-terminal part of the protein by using a D-amino acid composed peptide. Following the selection, the oligonucleotides were screened by filter binding assay, and surface plasmon resonance analysis of the most auspicious candidates demonstrated that this approach could provide spiegelmers with subnanomolar dissociation constant. To demonstrate if the selected spiegelmers are functional and suitable for cTnI detection in a sandwich type arrangement, AlphaLisa technology was leveraged and the obtained results demonstrated that spiegelmers with different epitope selectivity are suitable for specific detection of cTnI protein even in human plasma containing samples. These results suggest that spiegelmers could be considered in the development of the next generation cTnI monitoring assays.

## 1. Introduction

The significance of aptamers is increasingly appreciated by the scientific community and their diagnostic potential is also attested by a vast number of publication describing the development of aptamer-based biosensors [1]. The intense research interest in aptamers has also brought about commercially available human diagnostic tests for measuring the concentration of active thrombin and protein C [2,3]. These assays rely on the so-called oligonucleotide-based enzyme capture assay (OECA), that is, the protein of interest selective aptamer is immobilized on the plate and the captured protein is detected through its enzyme activity by using fluorogenic substrates. Notwithstanding, practical leveraging of aptamers in routine diagnostics is dishearteningly sporadic and no aptamer-based test has been approved for clinics yet.

The moderate infiltration of aptamers into clinical diagnostics might be explained by their susceptibility to the ubiquitously present nucleases that results in their rapid degradation in body fluids [4]. To evade this shortcoming, various modified nucleotide possessing aptamers of increased half-lives have been presented, but none of them are entirely nuclease resistant [5]. The only exceptions are the L-ribose or L-2′-deoxyribose units composed oligonucleotides, known as spiegelmers. These enantiomers of natural nucleic acids are completely unsusceptible to the prevailing nucleases, while their selectivity and affinity is comparable to those of aptamers [6]. Due to the size limitations of chemical peptide synthesis and inappropriate folding of chemically synthesized proteins, the main bottleneck of spiegelmer selection is the requirement of a mirror image of protein target. Consequently, most of the spiegelmers have been selected for small molecules, cytokines, and peptide hormones [7,8,9]. The only published spiegelmer that was isolated using a full-length D-enantiomer protein as target of SELEX (Systematic Evolution of Ligands by EXponential Enrichment) is selective for a small, 110 amino acid-composed RNase, indicating the limits of this approach [10]. Notwithstanding, the structural analysis of aptamer- and spiegelmer-protein complexes revealed that these oligonucleotides interact with their target through definite amino acid motifs; thus, theoretically protein-selective spiegelmers can be generated without application of D-enantiomers of complete proteins [11,12]. This so-called domain approach of spiegelmer selection follows the rationality of antibody production, i.e., only a peptide motif of the protein of interest is used for triggering the immune response [13]. In a similar manner, unique protein selective spiegelmers could be isolated by using an appropriately chosen peptide motif of the protein of interest as targets of selection. Previously, we further developed and successfully applied the domain method to produce spiegelmers for an N-terminally localized peptide motif of cardiac troponin I (cTnI), one of the generally accepted standard biomarkers of acute coronary syndrome (ACS) [14]. In the latter study, these spiegelmers were leveraged for developing an antibody-spiegelmer-composed homogenous sandwich assay that was suitable for selective detection of cTnI [15].

In the early days of biomarker-based diagnosis of ACS, necrosis of the heart muscle cells was monitored by measuring aspartate transaminase activity of blood samples; thus, the specificity of the measurement was ensured by the substrate selectivity of the enzyme [16]. The presently accepted biomarkers of ACS, the heart specific isoforms of troponin T and I, also known as cardiac troponins (cTns), do not possess enzyme activity; therefore, their selective detection strictly relies on affinity assays [17]. Currently, all clinically approved cTns measuring devices apply antibodies in sandwich assays of various detection methods. The sensitivity of these assays has been tremendously improved since the publication of the first ELISA of cTnT, i.e., the limit of detection has dropped from the ng/mL (~nM) to pg/mL (~pM) range [18]. The presently applied, so-called high-sensitivity cTns (hscTns) are even suitable for measuring the few pg/mL concentrations of cTns in healthy patients. This advancement leads to a more general leveraging of cTns assay; they have been introduced into the diagnostics of early acute myocardial infarction and chronic cardiomyopathies that are accompanied by milder plasma cTns level elevations [17].

**Figure 1:**
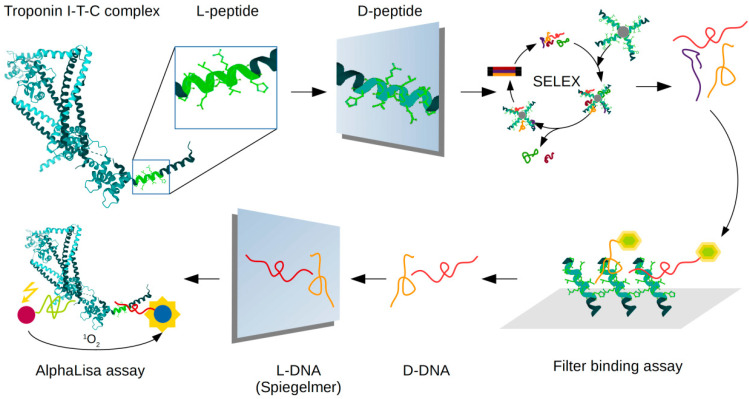
Schematics of spiegelmer-based sandwich assay development for cardiac troponin I detection. These proof of concept results provide the first example for fully spiegelmer-based sandwich assay of diagnostic potential as supported by their applicability for cTnI in blood plasma.

In this study, we show the selection of a novel cTnI specific spiegelmers by the domain approach. The proposed procedure is more straightforward and cost-effective in comparison to the previously published protocol and resulted in highly-selective spiegelmers with dissociation constants in the picomolar range. Our formerly published spiegelmer is specific for an N terminal peptide motif of cTnI, whereas the current selection process targets an epitope that is localized in the proximity of the carboxyl terminus of troponin. We show that the spiegelmers selected in the present study could be applied in a sandwich-type assay for selective detection of cTnI in pair with the previously reported spiegelmers (Figure 1).

## 2. Results

### 2.1. Selection of cTnI Specific Spiegelmers

Due to its phosphorylation, complex formation and rapid degradation, cTnI is a challenging analyte. Considering these constraints and the N-terminal epitope selectivity of our previously generated spiegelmer, a unique peptide motif corresponding to the positions 161–169 of cTnI was chosen as the target of selection. The D-enantiomer peptide of the assigned amino acid sequence was synthesized with a C-terminal cysteine extension and covalently linked onto autoreactive bromoacetyl paramagnetic particles. Prior to selection, negative SELEX was implemented, i.e., the initial single-stranded DNA library was challenged with unmodified paramagnetic beads to remove the bead-interacting oligonucleotides. The ssDNA (single-stranded DNA) library was enriched for peptide selective oligonucleotides by seven cycles of SELEX and the non-specifically interacting ssDNA were eliminated by a further counter-selection step using serum protein coated magnetic beads following the fourth cycle of selection. In order to enhance the affinity of the oligonucleotides, the selection pressure was gradually intensified by decreasing the peptide-coated particle concentration and incubation time and increasing the stringency of washing conditions. Finally, competent cells were transformed by vectors bearing PCR products of the last selection cycle and the nucleic acid sequence of vector inserts were determined by Sanger sequencing.

According to the theory of SELEX, the process results in the accumulation of target selective oligonucleotides of the highest affinity. However, in practice, SELEX is distorted by PCR bias; thus, not all the accumulated ssDNAs necessarily represent practically useful aptamers. Consequently, the individual oligonucleotides have to be experimentally evaluated to designate the ssDNAs possessing the best binding properties. To circumvent the costly synthesis of each selected spiegelmer candidate, we previously introduced a screening step with a reverse approach, i.e., the interaction of isolated D-oligonucleotides and D-cTnI peptide ligands was measured by surface plasmon resonance imaging (SPRi) [14]. In order to evade the SPRi demand of this approach, fluorescently labelled ssDNAs were produced by PCR and alkaline denaturation, and then incubated with the target peptide-coated beads. Following the washing steps, the beads were transferred onto nitrocellulose membrane and the peptide-bound oligonucleotides were detected by reading the fluorescence intensity.

**Figure 2:**
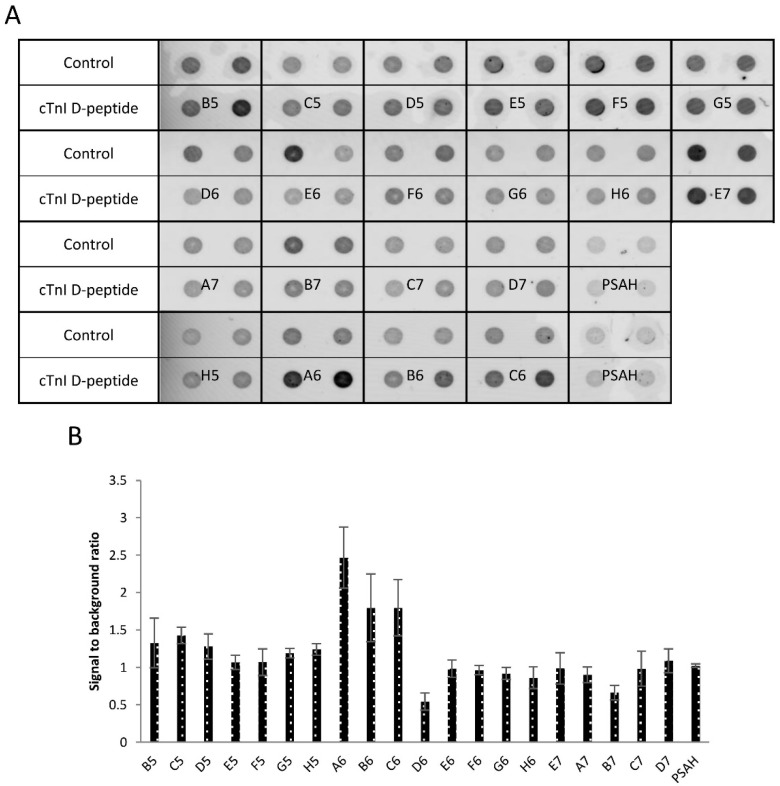
Screening of isolated oligonucleotides by filter binding assay. (**A**) Non-modified bead or 10 pmol of bead-bound cTnI D-peptide was incubated with 0.4 µM Cy5 labelled D-oligonucleotide solution. Following the washing steps, the beads were transferred onto nitrocellulose membrane and the fluorescence signal was detected by a fluorescence scanner. (**B**) Relative fluorescence intensities as result of the quantitative evaluation of the fluorescence images.

Although the obtained data indicated that most of the selected oligonucleotides produced higher fluorescence signal in comparison to an unrelated control aptamer, several of them were also bound to the bare magnetic particles thus were not suitable for specific detection of the peptide ligand (Figure 2A). The fluorescent signals were also quantitatively evaluated by densitometry and these results showed that A6, C6, and B6 oligonucleotides possessed the highest signal to background ratio (Figure 2B).

### 2.2. Functional Characterization of the Selected Spiegelmers

**Figure 3:**
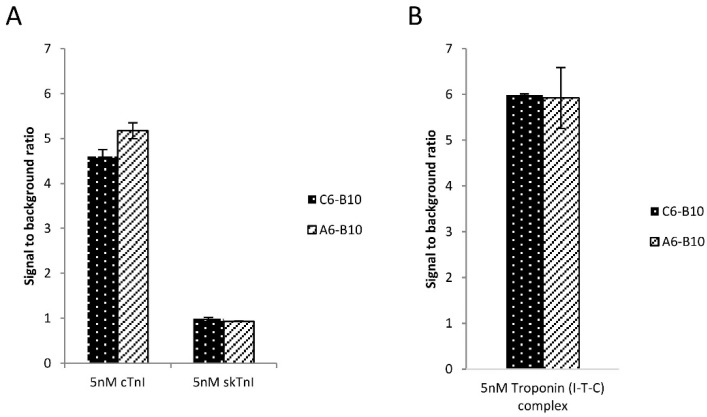
Selective detection of cTnI monomer and ternary complex by spiegelmer based sandwich assay. (**A**) Ten times diluted cTnI depleted human plasma was spiked with 5 nM recombinant cTnI and sTnI and analyzed by AlphaLisa in a dedicated plate reader using the N-terminal B10 and the C-terminal epitope selective A6, C6 spiegelmers. (**B**) The selection buffer was completed by the addition of 5 nM human I-T-C ternary complex and analyzed as in panel A.

Based on the filter binding assay results, two of the best performing oligonucleotides, A6 and C6 were chemically synthesized from L-nucleotides with a 5′ terminal biotin label (Supplementary Table S1). The presented spiegelmers were generated to develop a completely spiegelmer based cTnI detecting sandwich assay. Therefore, to study the functionality of these two spiegelmers, we set up an AlphaLisa assay by using streptavidin coated donor and acceptor beads, the previously selected N-terminal epitope specific B10 and the novel C6 and A6 spiegelmers. First, the B10 spiegelmer was mixed with diluted human plasma of 5 nM cTnI or sTnI concentration and, following 1-h incubation, the acceptor bead was also added to the mixture. Finally, the blend was complemented with either A6 or C6 pre-coated donor beads and the luminescence signal was read after a further incubation step. The collected data indicated that the signal intensity was increased approximately five times upon addition of cTnI in both arrangements indicating the utility of the novel spiegelmers (Figure 3A). Importantly, none of the spiegelmers produced increased luminescence with the sTnI containing mixture implying high selectivity of the sandwich assays. Upon necrosis of myocytes, cTnI is also released as a part of a ternary cTnT–cTnI–cTnC (I-T-C) complex; thus, the optimal ACS diagnostic assay is expected to detect both forms of cTnI [1].

Considering this requirement, we set out to further study the putative diagnostic applicability of the above described sandwich assays by using the ternary complex as the analyte of AlphaLisa. The streptavidin coated donor and acceptor beads were pre-incubated with B10 and A6 or C6 spiegelmers, respectively, and then incubated in the selection buffer containing 5 nM ternary troponin complex. Both spiegelmer arrangements demonstrated an increase in fluorescence signal upon addition of I-T-C by about six times (Figure 3B). These results indicated that the two novel spiegelmers are equally suitable for the detection of the monomeric and I-T-C complex form of cardiac troponin I.

In order to gain more detailed information about the troponin binding characteristics of our novel spiegelmers, kinetic analysis of the spiegelmer-ternary complex interactions by surface plasmon resonance imaging (SPRi) was implemented. The spiegelmers were microspotted onto Extravidin modified gold SPRi chip and challenged with I-T-C complex of different concentrations (Supplementary Figure S1). Evaluation of the multi-cycle kinetics showed that the equilibrium dissociation constants of both oligonucleotides were in the subnanomolar range (*K*_D_ = ~540 pM for A6 and ~305 pM for C6) (Supplementary Figure S2). These values indicate that the novel spiegelmers are superior to our previously generated B10 spiegelmer and the published cTnI selective aptamers [14,19].

Shortened aptamers are less expensive and easier to synthesize; furthermore, truncated oligonucleotides might have higher affinity and selectivity in comparison to their parental, full-length version [2]. Therefore, the primer region, excluding the variant of the slightly better affinity spiegelmer, C6, was synthesized and applied in AlphaLisa to reveal the effect of truncation on its functionality. Although the shortened spiegelmer remained functional, its binding capacity was significantly deteriorated; thus, our further studies were accomplished by applying the full-length C6 spiegelmer (Supplementary Figure S3A,B).

### 2.3. Determination of cTnI Concentration by Spiegelmer Sandwich Assay

**Figure 4:**
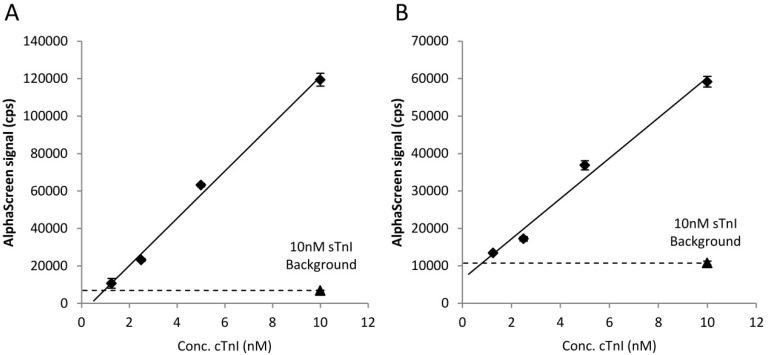
AlphaLisa signal of the sandwich assay as a function of the concentration of cTnI. The selection buffer (**A**) or diluted serum (**B**) was formulated with various amounts of recombinant cTnI or sTnI to reach the indicated final concentrations. The signals were measured by a fluorescence plate reader. The adjusted R-squared values were 0.9945 and 0.9843, respectively.

We implemented studies to reveal whether the developed sandwich assay is suitable for determination of cTnI concentration. To this end, the buffer of the spiegelmer selection was supplemented with a gradually increasing amount of recombinant cTnI or an appropriate amount of sTnI to reach 10 nM final concentration and the luminescence signal of AlphaLisa mixtures was monitored following the above described protocol. The collected signal adequately correlated with the cTnI concentration and was not increased in the sTnI spiked samples (Figure 4A). Next, we aimed to demonstrate the applicability of the sandwich assay in a complex protein matrix; thus, the same experiment was carried out, but the selection buffer was replaced with ten times diluted cTnI-free human blood plasma.

In harmony with the results of the ideal buffer containing measurements, the intensity of the obtained luminescence showed a good linearity in the ng/mL range of troponin concentration (Figure 4B). These data collectively indicate that the selected spiegelmers could be applied in sandwich assay for measuring cTnI concentration in even clinically relevant matrices.

## 3. Discussion

The spiegelmer technology was introduced by generating an L-RNA ligand that binds D-adenosine more than twenty years ago and since then, almost all spiegelmers have been selected against peptide targets [7]. Previously, we showed that protein-selective spiegelmers could be isolated without chemical synthesis of the complete D-enantiomer variant of the protein of interest by using a reasonably chosen peptide epitope as the target of selection [14]. We improved the domain method and demonstrated that spiegelmer pairs which are suitable for sandwich assay development also could be generated by following this rationale.

In order to increase the selectivity of enriched oligonucleotides, negative selection steps have been routinely applied since the early days of aptamers [20]. Although the negative selection significantly increases the success rate of aptamer generation, the presented results highlight that individual experimental characterization of enriched oligonucleotides cannot be evaded, since many of the isolated oligonucleotides would rather bind to the immobilization matrix than to the ligand itself. To circumvent the costly chemical synthesis of spiegelmers, we previously introduced a spiegelmer screening protocol that relied on SPR immobilized D-oligonucleotides and D-amino acid composed epitope of the target protein [14]. In the present publication, we show that this approach could be implemented in a reverse arrangement, i.e., the magnetic bead-bound target peptide of selection process is incubated with the fluorescently labelled oligonucleotides and the interaction is monitored following the transfer of the bead-bound peptide-oligonucleotide complex onto nitrocellulose membrane. This development lowers the equipment demand during the screening process and is more cost-effective and thus more amenable for laboratories of general instrumentation and molecular biology expertise.

The presented proof-of-principle study provides the first example of a fully spiegelmer based diagnostic sandwich assay. The obtained results demonstrate that spiegelmers can warrant the needed selectivity and detect various forms of cTnI even in such complex matrices as blood serum. The currently available high-sensitivity cTnI measuring kits could quantitatively detect pg/mL concentrations of analyte and thus clearly outperform the presented spiegelmer sandwich assay. Of note, the monoclonal antibodies of high sensitivity cTnI diagnostic kits possess K_D_ values in the 74 nM to 2.6 μM range that is approximately two magnitudes higher than those of our spiegelmers [21]; thus, the relatively low sensitivity of our sandwich assay is most likely not attributable to the low performance of spiegelmers. Detection limit of the only commercially available cTnI detecting AlphaLISA kit is under 10 pg/mL implying high sensitivity of the approach. Although dissociation constants of applied antibodies of the kit are not provided, they are most likely inferior to those of the here presented spiegelmers. Furthermore, it is known that the limit of detection of AlphaLISA is determined by many factors, such as buffer composition, concentration of receptors, and acceptor and donor beads, and even the order of the addition of various components may affect the assay sensitivity [22]; thus, the low pg/mL sensitivity of the kit has been achieved by thorough optimization of assay conditions

Considering that the development of antibody-relying hs-cTnI assays demanded decades, it is expected that the application of detection methods of higher sensitivity and assay optimization will make a similar detection range achievable by using spiegelmers [23]. In view of the presented results and the advantageous chemical characteristics of spiegelmers, these nuclease resistant oligonucleotides should be considered as plausible receptors of diagnostic devices.

## 4. Material and Methods

### 4.1. Random Oligonucleotide Library and Primers

The random oligonucleotide library was comprised of a central random region of 38 nucleotides (N38) with fixed flanking sequences: 5′-CAG TGA GTG ATG GTG AGG G-N38-CCC ACA CTG TCC ATA CAC G-3′ (Integrated DNA Technologies). 5′-CAG TGA GTG ATG GTG AGG G-3′ forward primer and 5′ biotin labeled 5′-CGT GTA TGG ACA GTG TGG G-3′ reverse primer were used in SELEX. The most promising sequences were chemically synthesized from L-nucleotides with 5′ biotin labeling (IBA GmbH, Göttingen, Germany) (Supplementary Table S1).

### 4.2. Selection of cTnI Specific Spiegelmers

A C terminal peptide motif of cTnI protein was extended by a cysteine for thiol-based crosslinking (RAKESLDLRA-C) and synthesized from D-amino acids (JPT Peptide Technologies, Berlin, Germany). The target peptide was covalently bound to bromoacetyl paramagnetic beads according to the manufacturer instructions (Chemicell, Berlin, Germany).

The 1. nmol random oligonucleotide library was incubated 1 h at RT with mild shaking with 25 µL blocked bromoacetyl paramagnetic particles in PBS to eliminate the paramagnetic bead binding oligonucleotides (counter-selection). In the first selection cycle, the counter-selected oligonucleotide library was incubated 1 h at RT with mild shaking with 500 pmol paramagnetic beads bound D-cTnI peptid in 2 mL selection buffer (1 mg mL^−1^ BSA; 0.01 µg mL^−1^ poly (dI-dC); 5 mM EDTA in PBS). Magnetic beads were washed 3 times with 100 µL PBS then the D-cTnI peptide bound oligonucleotides were heated to 95 °C and eluted in 32 µL UltraPure Distilled Water (Invitrogen, Carlsbad, CA, USA). The selection cycles were repeated 7 times (Supplementary Table S2) by gradually decreasing the amount of magnetic particle-bound peptide and the incubation time, whereas dI-dC concentration of the binding buffer and stringency of washing conditions were increased (Supplementary Table S2). Prior to the fifth selection step, selectivity of oligonucleotides was also enhanced by introducing a further counter-selection step against serum proteins, i.e., the enriched oligonucleotide library was incubated for 1 h with 400 µg of SiMag-Cyanuric magnetic beads (Chemicell, Berlin, Germany) coupled cTnI-free serum proteins (Hytest, Turku, Finland).

Amplification of enriched oligonucleotides was carried out by emulsion PCR (EURx, Gdansk, Poland). The 50 µL of PCR mixtures contained 10 µL 5× HF reaction buffer, 0.4 U of iProof polymerase (Bio-Rad), 500 nM forward primer, 500 nM biotinylated reverse primer, 0.4 mM CleanAmp dNTP (Trilink, San Diego, CA, USA), and 10 µg mL^−1^ BSA. Water in oil emulsion was prepared according to the manufacturer’s instructions. The amplification conditions were: 5 min denaturation at 95 °C, 25 cycles of 10 sec denaturation at 95 °C, 10 sec annealing at 59 °C, and a 10 sec polymerization step at 72 °C. Final extension step was performed at 72 °C for 2 min. The success of the amplification was monitored by 10% polyacrylamide gel electrophoresis.

To regenerate the single stranded DNA pool for the next selection cycle, 50 μL PCR product was supplemented with equal volume of 2× bind and wash buffer (B&W, 20 mM Tris-HCl (pH 7.5), 2 mM EDTA, 4 M NaCl), and incubated with 10 μL Dynabeads MyOne C1 Streptavidin beads (Thermo Fischer Scientific) for 30 min, then washed with 3 x 200 μL of 1x B&W buffer. Biotinylated reverse and non-biotinylated sense DNA strands were separated by 10 min incubation in 20 μL of 20 mM NaOH and the supernatant was neutralized by addition of 3 μL of 200 mM NaH_2_PO_4_.

Following the last SELEX cycle, the PCR products were ligated into Zero Blunt™ TOPO™ vector by PCR Cloning Kit (Thermo Fischer Scientific, Waltham, MA, USA) and transfected into α-select Gold competent cells (Bioline, London, UK). Colony PCR was accomplished by using the common M13 primers and 20 products of the correct size were sequenced with Sanger method.

### 4.3. Screening of Oligonucleotides by Filter Binding Assay

The Cy5 labelled ssDNAs were produced by PCR. Briefly, PCR mixtures contained 25 µL 2x PCR BIOTaqMix (PCR Biosystems, London, UK) PCRBIO Taq DNA Polymerase, 6 mM MgCl_2_, 2 mM dNTPs, 500–500 nM 5′- Cy5 labelled forward and 5′- biotinylated reverse primers of the oligonucleotide library. Diluted colony PCR products were applied as templates of amplification. The PCR consisted of 1 min denaturation at 95 °C, 30 cycles of 10 sec denaturation at 95 °C, 10 sec annealing at 59 °C, and a 10 sec polymerization step at 72 °C. Final extension step was 72 °C for 2 min. The success of the amplification was monitored by electrophoresis using 3% agarose gel. 5′- Cy5 labelled ssDNA was generated by alkali denaturation as described above. The purity of the ssDNA samples was monitored by 10% polyacrylamide gel electrophoresis.

Nitrocellulose membrane (Bio-Rad, Hercules, CA, USA) was pre-treated with 400 mM KOH for 10 min and rinsed with PBS. Cy5 labeled oligonucleotides were mixed with 10 pmol bead-bound cTnI peptide at various concentrations in 50 μL PBS buffer. Following 1-h incubation at RT, the magnetic particles were washed 3 times with 100 uL PBS and transferred onto the nitrocellulose membrane by using a dot-blot apparatus with 20 mbar vacuum. The membrane was scanned at 633 nm excitation 670 nm emission wavelength with Typhoon 9410 Imager. The images were analyzed with the ImageQuant software.

### 4.4. Microspotting of Spiegelmers on Surface Plasmon Resonance Imaging (SPRi) Chips

Extravidin modified (CSe) gold SPRi biochips (HORIBA FRANCE SAS, Palaiseau, FranceCountry) were stored under an argon atmosphere at 4 °C in a refrigerator, protected from light. The immobilization of the biotinylated L-DNA spiegelmers were made by microspotting using a BioOdyssey™ Calligrapher™ miniarrayer (Bio-Rad) with a 500 µm diameter SMP15 Stealth Micro Spotting Pin (Arrayit Corporation, Sunnyvale, CA, USA) having an uptake volume of 0.25 μL. The biotinylated L-DNA spiegelmers were formulated in PBS at different concentrations (between the range of 0.625–10 µM) and aliquots of 20 µL were loaded into the wells of a DNA LoBind, PCR clean, 384 well LD-PE plate (Eppendorf, Hamburg, Germany). The spotting chamber was adjusted to 65 rh% with the spotting stage thermostated at 12 °C and an automatic spotting sequence deposited 3 parallel spots onto the SPRi chip surface at each biotinylated spiegelmer concentration level. The spotted microarrays were then incubated at 20 ± 1 °C and 65 rh% for 12 h. In these conditions, the drying of the spotted droplets was avoided during surface modification and the droplets were still visible before the L-DNA chips were blocked with 10 µg/mL biotin in phosphate buffer saline (PBS,) for 30 min and then with 0.5 mM (11-mercaptoundecyl)tetra(ethylene glycol) (Sigma-Aldrich, St. Louis, MO, USA) in PBS for 30 min. Finally, the chip was washed with DI water and gently dried under N_2_ stream.

### 4.5. SPRi Measurements

For surface plasmon resonance imaging measurements, a HORIBA XelPleX SPRi system (HORIBA FRANCE SAS) was used at fixed optimal angles. First, the working angles (typically 3 angles) were selected based on the recorded SPR reflectivity curves (reflectivity vs. incident angle) to measure the interactions of the spiegelmers with cTnI-T-C target molecules. The reflectivity response (refractive index sensitivity) over the whole chip surface was normalized by using 3 mg mL^−1^ sucrose solution. The binding of the troponin complex to the spiegelmer probes was monitored upon injecting 500 µL target aliquots in PBS working buffer (10 mM phosphate buffer, 137 mM NaCl, 2.7 mM KCl, pH 7.4) at 25.0 °C, at a flow rate of 50 µL min^−1^. The samples were preloaded in the wells of a 96 deep well Protein LoBind LD-PE plate (Eppendorf, Hamburg, Germany) placed into the auto sampler unit of the instrument thermostated at 7 °C. Between consecutive target-spiegelmer interaction measurements, the surface-confined L-DNA probes were regenerated by 200 µL of 100 mM NaOH solution for 4 min. The typical durations for recording the baseline, association and dissociation steps were 1, 10, and 5 min, respectively. The binding kinetics were determined with the Scrubber 2 GenOptics version software (BiaLogic Software).

### 4.6. AlphaLisa Measurements

AlphaLisa sample mixtures were assembled in 384 well plate (AlphaPlate−384 SW, Perkin Elmer, Waltham, MA, USA). 10 nM final concentration of biotin labelled N-terminal epitope specific spiegelmer (B10) was incubated with varying amount of wheat-germ in vitro translated GST-tagged cardiac troponin I protein (1.25, 2.5, 5, 10, and 60 nM) [24] or human troponin ITC complex (5 nM) (HyTest, Cat.# 8T62, Turku, Finland) in 20 µL PBS completed with 1 mg mL^−1^ BSA and 0.1 µg mL^−1^ poly (dI-dC) at RT for 1 h. Human skeletal troponin I (Hytest, Cat.# 8T25) was used as negative control. Spiegelmer sandwich type assay was also examined in 10 times diluted Troponin I-free serum (Hytest, Cat.# 8TFS) supplemented buffer. AlphaLisa Streptavidin Acceptor beads (20 µg mL^−1^) (Perkin Elmer) were added to each well. 10nM final concentration of biotin labelled C-terminal epitope specific spiegelmer (A6 or C6 or C6sh) was incubated separately with AlphaScreen Streptavidin Donor beads (20 µg mL^−1^) (Perkin Elmer, Waltham, MA, USA). Following 30 min incubation at RT, the AlphaLisa Acceptor and Alpha Screen Donor bead containing fraction were mixed and incubated 2 additional hours at 25 °C temperature before the luminescence signal detection in an EnSpire multi-mode plate reader (Perkin Elmer).

## Supplementary Materials

Supplementary materials can be found at https://www.mdpi.com/1422-0067/21/14/4963/s1. Supplementary Table S1: L-Nucleic acid sequences of the studied spiegelmers; Supplementary Table S2: Conditions of SELEX cycles; Supplementary Figure S1: Differential SPR image of spiegelmer microarray microspotted in triplicate from various concentration of L-DNA probe solutions (0.625-10 µM). The color of the spots changes with the amount of bound cTnI-T-C protein, as shown on the scale on the right; Supplementary Figure S2: Interaction curves upon injection of different concentration of the target protein (9.6, 19.2, 38.4 nM) recorded for C6 (left) and A6 (right) spiegelmer spots immobilized at 2.5 µM. Kinetic curves were fitted one by one to each parallel spot (3 replicates) on the same SPRi chip; Supplementary Figure S3: The effect of spiegelmer truncation on the sandwich type assay. Selection buffer (A) or ten times diluted human plasma (B) was spiked with 5 nM ternary complex and analyzed by AlphaLisa.
