# Supplementary material for: Spiegelmer-Based Sandwich Assay for Cardiac Troponin I Detection
Source: Int J Mol Sci. 2020 Jul 14;21(14):4963. doi: 10.3390/ijms21144963 (PMC7404307; doi:10.3390/ijms21144963)
Supplement: Supplementary file 1 [file ijms-21-04963-s001.pdf]

SUPPLEMENTARY DATA

Spiegelmer-based sandwich assay for cardiac troponin I Detection

Zoltán János Tolnai [1], Judit András [1], Zsuzsanna Szeitner [1], Krisztina Percze [1], László Ferenc Simon [2], Róbert E. Gyurcsányi [2] and Tamás Mészáros[2,*]

[1] Department of Medical Chemistry, Molecular Biology and Pathobiochemistry, Semmelweis University, Tűzoltó u. 37-47., H-1094, Budapest, Hungary;

[2] BME "Lendület" Chemical Nanosensors Research Group, Department of Inorganic and Analytical Chemistry, Budapest University of Technology and Economics, Szt. Gellért tér 4, H-1111 Budapest, Hungary

* Correspondence: meszaros.tamas@med.semmelweis-univ.hu

Supplementary Table 1. L-Nucleic acid sequences of the studied spiegelmers.

Name	Sequence
B10	5'-AGT CTC CGC TGT CCT CCC GAT GCA CTT GAC GTA TGT CTC ACT TTC TTT TCA TTG ACA TGG GAT GAC GCC GTG ACT G-3'
A6	5'-CAG TGA GTG ATG GTG AGG GCT TAG TTC GCC GCT CAT GCC GAA TCT CCT GTA TAA ATA CCC ACA CTG TCC ATA CAC G-3'
C6	5'-CAG TGA GTG ATG GTG AGG GTG AAT CGG TGT CGA CTA TTA AAT TAA GTT GTG GTT GTT CCC ACA CTG TCC ATA CAC G-3'
C6 short	5'-TG AAT CGG TGT CGA CTA TTA AAT TAA GTT GTG GTT GTT-3'

Supplementary Table 2. Conditions of SELEX cycles**.**

SELEX cycle number	Amount of target molecules	Incubation time	Buffer composition	Washing steps
1. Counter-SELEX	-	1 hour	2 mL PBS	-
1.	500 pmol cTnI peptide	1 hour	Supernatant of the 1. counter-SELEX<br>1 mg mL^{-1} BSA<br>0.01 µg mL^{-1} poly (dI-dC)<br>5mM EDTA	3x100 µL PBS
2.	375 pmol cTnI peptide	30 min	2 mL PBS<br>1 mg mL^{-1} BSA<br>0.01 µg mL^{-1} poly (dI-dC)<br>5mM EDTA	3x100 µL PBS
3.	375 pmol cTnI peptide	30 min	2 mL PBS<br>1 mg mL^{-1} BSA<br>0.1 µg mL^{-1} poly (dI-dC)<br>5 mM EDTA	3x100 µL PBS
4.	250 pmol cTnI peptide	30 min	2 mL PBS<br>1 mg mL^{-1} BSA<br>0.1 µg mL^{-1} poly (dI-dC)<br>5 mM EDTA	3x5 min, 100 µL PBS
2. Counter-SELEX	400 µg cTnI free serum proteins	1 hour	2 mL PBS<br>1 mg mL^{-1} BSA	
5.	250 pmol cTnI peptide	30 min	Supernatant of the 2. counter-SELEX<br>0.1 µg mL^{-1} poly (dI-dC)<br>5 mM EDTA	3x5 min, 100 µL PBS
6.	250 pmol cTnI peptide	15 min	2 mL PBS<br>1 mg mL^{-1} BSA<br>0.1 µg mL^{-1} poly (dI-dC)<br>5 mM EDTA	3x5 min, 100 µL PBS
7.	250 pmol cTnI peptide	15 min	2 mL PBS<br>1 mg mL^{-1} BSA<br>0.1 µg mL^{-1} poly (dI-dC)<br>5 mM EDTA	3x5 min, 100 µL PBS, 0.3 mM dextran sulfate

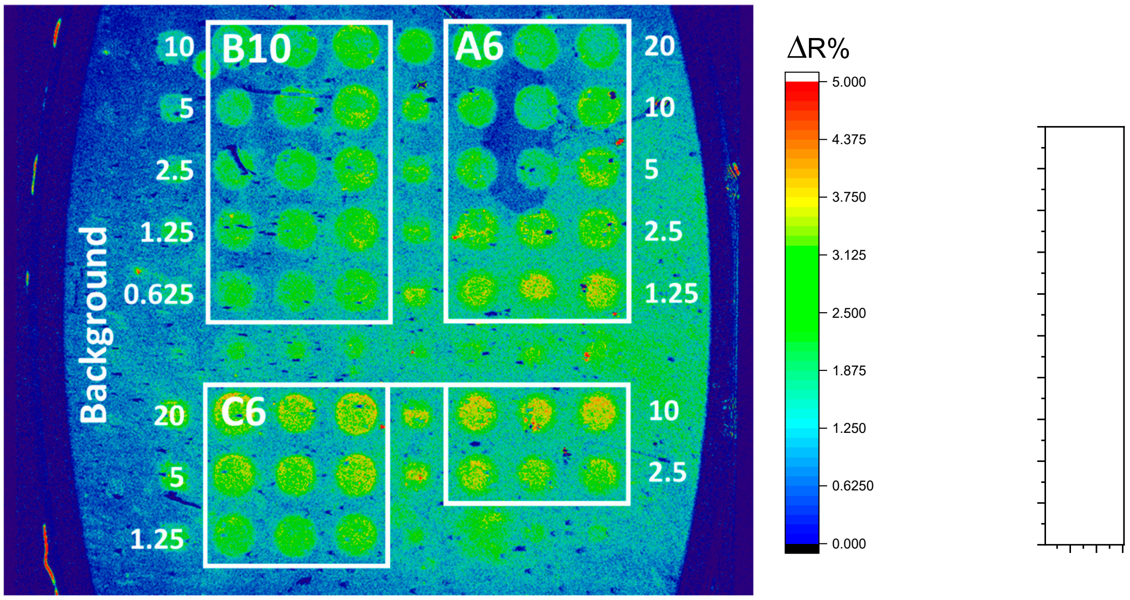

Supplementary Figure 1. Differential SPR image of spiegelmer microarray microspotted in triplicate from various concentration of L-DNA probe solutions (0.625-20 µM). The color of the spots changes with the amount of bound cTnI-T-C protein, as shown on the scale on the right.

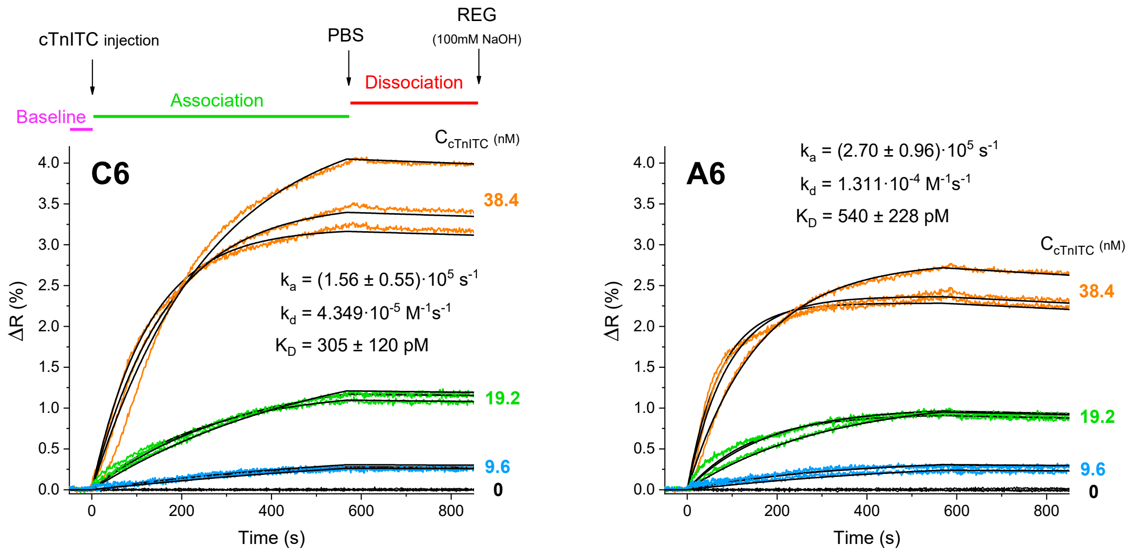

Supplementary Figure 2. Interaction curves upon injection of different concentration of the target protein (9.6, 19.2, 38.4 nM) recorded for C6 (left) and A6 (right) spiegelmer spots immobilized at 2.5 µM. Kinetic curves were fitted one by one to each parallel spot (3 replicates) on the same SPRi chip.

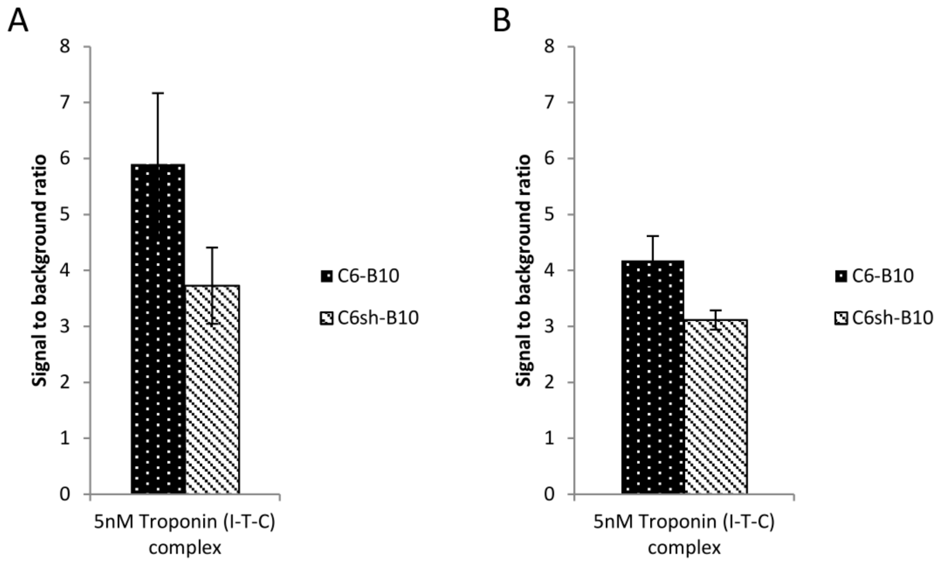

Supplementary Figure 3: The effect of spiegelmer truncation on the sandwich type assay. Selection buffer (A) or ten times diluted human plasma (B) was spiked with 5 nM (385 ng mL^{-1}) ternary complex and analyzed by AlphaLisa.
